# Three alternative splicing variants of *Loquacious* play different roles in miRNA- and siRNA-mediated RNAi pathways in *Locusta migratoria*

**DOI:** 10.1080/15476286.2023.2223484

**Published:** 2023-06-13

**Authors:** Yanli Wang, Huiyong Li, Xiaojian Liu, Lu Gao, Yunhe Fan, Kun Yan Zhu, Jianzhen Zhang

**Affiliations:** a Institute of Applied Biology, Shanxi University, Taiyuan, Shanxi, China; b College of Life Science, Shanxi University, Taiyuan, Shanxi, China; c Department of Entomology, 123 Waters Hall, Kansas State University, Manhattan, KS, USA; d Key Laboratory of Chemical Biology and Molecular Engineering of Ministry of Education, Shanxi University, Taiyuan, China

**Keywords:** *Locusta migratoria*, Loquacious, exo-siRNA pathway, endo-siRNA pathway, miRNA pathway

## Abstract

RNA interference (RNAi) is a specific post-transcriptional gene-silencing phenomenon, which plays an important role in the regulation of gene expression and the protection from transposable elements in eukaryotic organisms. In *Drosophila melanogaster*, RNAi can be induced by microRNA (miRNA), endogenous small interfering RNA (siRNA), or exogenous siRNA. However, the biogenesis of miRNA and siRNA in these RNAi pathways is aided by the double-stranded RNA binding proteins (dsRBPs) Loquacious (Loqs)-PB, Loqs-PD or R2D2. In this study, we identified three alternative splicing variants of *Loqs*, namely *Loqs-PA*, -*PB*, and -*PC* in the orthopteran *Locusta migratoria*. We performed *in vitro* and *in vivo* experiments to study the roles of the three Loqs variants in the miRNA- and siRNA-mediated RNAi pathways. Our results show that Loqs-PB assists the binding of pre-miRNA to Dicer−1 to lead to the cleavage of pre-miRNA to yield matured miRNA in the miRNA-mediated RNAi pathway. In contrast, different Loqs proteins participate in different siRNA-mediated RNAi pathways. In exogenous siRNA-mediated RNAi pathway, binding of Loqs-PA or LmLoqs-PB to exogenous dsRNA facilitates the cleavage of dsRNA by Dicer−2, whereas in endogenous siRNA-mediated RNAi pathway, binding of Loqs-PB or Loqs-PC to endogenous dsRNA facilitates the cleavage of dsRNA by Dicer−2. Our findings provide new insights into the functional importance of different Loqs proteins derived from alternative splicing variants of *Loqs* in achieving high RNAi efficiency in different RNAi pathways in insects.

## 1. Introduction

RNA interference (RNAi) is a highly conserved posttranscriptional gene silencing mechanism, which plays an important role in gene function, protection from transposable elements, and defence from viral infection in most eukaryotic organisms [1]. Certain small RNAs can trigger the RNAi machinery, which leads to the degradation of complementary RNA molecules in a sequence-specific manner [2,3]. According to the types of small RNAs that cause posttranscriptional silencing of target genes and their interacting proteins, the RNAi pathways can be divided into siRNA (small interfering RNA)-, miRNA (microRNA)- and piRNA (piwi interacting RNA)-mediated RNAi pathways [4]. The processes of both the siRNA-mediated and miRNA-mediated RNAi pathways require the assistance of double-stranded RNA binding proteins (dsRBDs). The main function of dsRBDs is to recognize and bind dsRNA and then determine the structure of dsRNA to make it enter the siRNA- or miRNA-mediated RNAi pathway. The siRNA-mediated RNAi pathway can be triggered by either endo-siRNAs generated from the transposon for genome transposon defence or exo-siRNAs generated from RNA viruses for viral defence or from experimentally introduced long dsRNAs [5]. These differences are important because the biogenesis and processing of miRNAs, endo-siRNAs and exo-siRNAs depend on different dsRBPs as Dicer binding partners [6,7]. Previous studies have shown that Loquacious (Loqs) and R2D2 [dsRNA-binding protein with two dsRNA-binding domains (R2) and ability to associate to Dicer−2 (D2)] are the dsRNA binding proteins involved in the RNAi pathways [8].

Loqs was first identified in *Drosophila*, which encoded a protein highly similar to the human TAR RNA binding protein (TRBP) [9]. In *Drosophila*, *Loqs* gene generates four distinct isoforms known as Loqs-PA, -PB, -PC and -PD [10,11]. The Loqs-PA and Loqs-PB interact with Dicer−1 during miRNA biogenesis, Loqs-PD interacts with Dicer−2 in endo-siRNA biogenesis, whereas the function of Loqs-PC is still unknown [12,13]. R2D2 is another dsRNA binding protein, whose roles are to assist Dicer−2 to stabilize exo-dsRNA and bind Dicer−2 to form a Dicer−2/R2D2 complex [14]. The complex assists the delivery of the siRNA duplex to Ago2, which is essential for RISC loading [14]. To date, however, the interactions among R2D2, Dicer−2 and Loqs have not been clearly elucidated. In *Drosophila*, study has shown that DmR2D2 and DmLoqs-PD competitively bind DmDicer−2 and play independent roles in exo-siRNA and endo-siRNA pathways, respectively [9]. Other studies proposed a model of sequential action where Loqs plays an important role in assisting dsRNA processing, whereas R2D2 is essential for subsequent loading of siRNAs into the effector RISC complex [15]. In *Aedes aegypti*, alternative splicing of *Loqs* mRNA results in three different isoforms, namely Loqs-PA, Loqs-PB, and Loqs-PC. Loqs-PA plays a complex role in both miRNA-mediated silencing and endo-siRNA pathway, which is important for the production of some siRNAs in coordination with R2D2 [16].

Although Loqs has been identified in many insect species, the function of their different isoforms in RNAi pathways has only been studied in *D. melanogaster* and *A. aegypti*. In particular, it is still unclear whether and how Loqs participates in exo-siRNA pathway. *Locusta migratoria* is an important agricultural pest and a model insect for studying various mechanisms of RNAi pathways. At present, however, there has been only little information about Loqs proteins in *L. migratoria* and their roles in various small RNA pathways are unclear. To determine the roles of dsRBP-Loqs in the small RNA pathways, we identified three different isoforms of *LmLoqs* based on the *L. migratoria* transcriptome and genome databases [17]. Our *in vitro* and *in vivo* studies have shown that the isoform LmLoqs-PA and LmLoqs-PB are involved in exo-siRNA mediated RNAi pathway, whereas the isoform LmLoqs-PB and LmLoqs-PC participate in endo-siRNA mediated RNAi pathway. In addition, we have found that LmLoqs-PB contributes to miRNA mediated RNAi pathway. These results indicate that the dsRBP-Loqs play an important role in siRNA-mediated and miRNA-mediated RNAi pathways in *L. migratoria*. These results have provided new knowledge on dsRBPs in small RNA pathways in insects. Better understanding of the RNAi pathways may help develop new strategies for improving RNAi efficiency for insect pest management.

## 2. Materials and methods

### 2.1. Insect rearing

The eggs of *L. migratoria* were incubated at the temperature of 30 ± 2°C, humidity of 60 ± 5%, and photoperiod of 14:10 h (light/dark) at the Institute of Applied Biology, Shanxi University, Taiyuan, China. After the eggs hatched, the nymphs were moved to a cage (50 cm x 50 cm x 50 cm) with a soft brush and fed fresh wheat seedlings and wheat bran every day.

### 2.2. Identification of the full-length cDnas of LmLoqs

The putative *LmLoqs* cDNA sequences were retrieved from the *L. migratoria* transcriptome and genome databases by bioinformatics methods using the sequence of *DmLoqs* [17]. Total RNA was extracted from the 3-day-old third-instar nymphs and 1 μg total RNA was used for the first-strand cDNA synthesis with oligo-(dT)18 primer (Sangon Biotech, Shanghai, China) by using M-MLV reverse transcriptase (TaKaRa, Kusatsu, Japan). The full-length cDNAs were then confirmed by PCR amplification from the first-strand cDNA template using the gene-specific primers (Table S1) followed by Sanger sequencing (Sangon Biotech, Shanghai, China).

The LmLoqs amino acid sequences were deduced from their confirmed full-length cDNAs using the translation program of ExPASy website (http://www.expasy.org/tools/dna.html). Domain structures were analysed using the website of SMART (http://smart.embl.de/). Multiple alignments of the deduced LmLoqs amino acid sequences were conducted by using ClustalX version 1.81 software. A neighbour-joining tree was constructed with 1000 bootstrap replicates in MEGA7 software [18]. The GenBank accession numbers are listed in Table S2.

### 2.3. Tissue- and development-specific expression patterns of *LmLoqs*

To analyse the tissue-specific expression pattern of *LmLoqs*, nine tissue samples, including integument, foregut, midgut, hindgut, Malpighian tubules, gastric caeca, fat bodies, testis, and ovary, were dissected from 3-day-old third-instar (N3D3) nymphs for total RNA extraction. To analyse the developmental expression patterns of *LmLoqs*, 1-day to 5-day-old third-instar nymphs of *L. migratoria* were collected for total RNA extraction. Each sample was prepared from four nymphs and each tissue type or developmental stage was analysed with three biological samples. The extraction of total RNA and the preparation of cDNA are described in Section 2.2. To ensure that the cycle threshold (Ct) was within an appropriate range (approximately 18–19), each cDNA sample was diluted 5-fold for reverse transcription quantitative PCR (RT-qPCR) analysis.

The specific primers used for RT-qPCR analyses of the *β-actin* and *LmLoqs* (Table S1) were designed using the software Primer Premier 5.0. RT-qPCR was performed using SYBR™ Green Real-time PCR Master Mix (Promega, Madison, WI, USA). A 20-μL reaction mixture contained 10 μL SYBR_®_ Green Realtime PCR Master Mix, 2 μL cDNA template, 0.8 μL (10 μM) each of forward and reverse primers, and 6.4 μL DEPC-treated water. RT-qPCR was performed using LightCycle ® 480 instrument II (Roche, Indianapolis, IN, USA). The RT-qPCR protocol consisted of 94°C for 60 s, followed by 40 cycles of 94°C for 5 s and 60°C for 31 s. Melting curves were used to assess the amplification specificity in each reaction and detect the presence of primer dimers.

### 2.4. RNAi of RNAi experiment

According to the cDNA sequences of *LmLoqs*, *Lmβ-Tubulin*, *LmLgl* (lethal giant larvae), and *GFP* (green fluorescent protein), the specific primers for the synthesis of each dsRNA were designed as shown in Table S1. The dsRNAs for *LmLoqs* (targeting the common region of all the three isoforms shown in the green box in Figure S1), *Lmβ-Tubulin*, *LmLgl* and *GFP* were synthesized using HiScribeTM T7 RNA High Yield RNA Synthesis Kit (New England Biolabs, Ipswich, MA, USA) and examined using 1% agarose gel to confirm the identity of each dsRNA. For RNAi experiments, 1-day-old third-instar nymphs were selected and 3 μg of *LmLoqs* dsRNA was injected into the haemocoel between the second and third ventral segments with a microinjector. The control group was injected with the same amount of ds*GFP*. After 48 h, the same dose of ds*Lmβ-Tubulin* or *LmLgl* was injected (e.g. ds*LmLoqs*+ds*Lmβ-Tubulin* or ds*LmLoqs*+ds*LmLgl*; ds*LmGFP*+ds*Lmβ-Tubulin* or ds*LmGFP*+ds*LmLgl*). Each group had six biological replicates and each biological replicate consisted of three nymphs. Each biological replicate was analysed with one technical replicate. The methods for the extraction of total RNA, reverse transcription, and RT-qPCR were the same as described in Section 2.3.

### 2.5. Generations of cDnas for truncated LmLoqs-PA/-PC proteins

The cDNA encoding a truncated LmLoqs-PA protein named LmLoqs-PA-mut was PCR-amplified from the correct vector (LmLoqs-PA-PGEM-Teasy) using the specific primers (Table S1). The cDNA encoding a truncated LmLoqs-PC protein named LmLoqs-PC-mut was synthesized by Sangon Biotech. Both the cDNAs were ligated to pGEM®-T Easy Vector (Promega) for the verification of their sequences.

### 2.6. Luciferase assays

To further clarify which isoform of LmLoqs was involved in exo-siRNA pathway, luciferase assays were performed. The coding sequences of the *LmLoqs-PA*, *LmLoqs-PB*, *LmLoqs-PC*, *LmLoqs-PA-mut*, and *Lmβ-Tubuli*n were inserted into pAc5.1 (Thermo Fisher, Waltham, MA, USA) expression vector and amplified as described in Section 2.2. S2 cells were cultured in a 24-well plate at 28°C until the cell concentration reached to 2 × 10^5^ cells/mL. Lipofectamine® 3000 (Invitrogen, Waltham, MA, USA) was used as a transfection reagent. Specifically, 0.75 μL Lipofectamine™ 3000, 2 μL P3000™, 0.5 μg psicheck−2 fluorescence vector and 1 μg pAc5.1 vector with expression of LmLoqs-PA/, -PB/, -PC/, -PA-mut; or Lmβ-Tubulin were mixed for 15 min and transfected S2 cells in each well. After 4 h, 2 μg ds*Luc* was added to S2 cells. The same amount of ds*Luc* was added to the control group. After incubation of 40 h, double-luciferase reporter assay system (Promega) was used to measure the firefly and *Renilla* luciferase activities in the Turner biosystems instrument (Promega). Western blotting was used to confirm whether the recombinant plasmids pAc5.1-LmLoqs-PA, pAc5.1-LmLoqs-PB and pAc5.1-LmLoqs-PC were expressed in S2 cells. The LmLoqs polyclonal antibody produced in rabbit by the ChinaPeptides Co., Ltd (CHN) was used as the verification antibody, whereas the anti-mouse β-Actin monoclonal antibody (Bioworld, Visalia, CA, USA) was used as an endogenous control.

To further determine which isoform was involved in endo-siRNA pathway, the target site of esi−2.1 was inserted into the downstream of the *hRluc* gene of psicheck−2. A pair of oligonucleotide (5’- GGAGCGAACTTGTTGGAGTCA−3’) containing two perfect-binding sites for *esi−2.1* were annealed and subcloned into psicheck−2 (*XhoI*/*NotI*) to generate psicheck−2-esi−2.1 [19]. One microgram of the constructed plasmids of pAc5.1-LmLoqs-PA/, -PB/, -PC/, -PC-mut; or pAc5.1-Lmβ-Tubulin (control) was transferred into each well of the S2 cells. After 4 h, 0.5 μg psicheck−2-esi−2.1 plasmid was transferred into each well. After the cells were cultured for 40 h, double luciferase reporter assay (Promega) was used to detect luciferase activity in Turner biosystems (Promega). Four biological replicates were used for each experimental group and the control group, respectively.

### 2.7. *In vitro* binding assay of LmLoqs protein and dsRNA

Each of the coding sequences of the *LmLoqs-PA*, *LmLoqs-PB*, *LmLoqs-PC* and *LmLoqs-PA-mut* was inserted into pET32a expression vector and amplified as described in Section 2.6. After *E. coli* BL21 (DE3) competent cells were transformed by the recombinant plasmid, they were cultured overnight at 37°C in solid Luria-Bertani (LB) medium containing ampicillin. The positive colonies were picked up and cultured in liquid LB media at 37°C. When the optical density reached at the range of 0.4 ~ 0.6, the cultures of the experimental group were induced with 0.2 mM IPTG at 16°C for 20 h, whereas the culture of the control group was cultured without IPTG. After ultra-sonication and centrifugation, the expressed proteins were resuspended in PBS.

Biotin-labelled ds*Lmβ-tubulin* was synthesized using HiScribeTM T7 RNA High Yield RNA Synthesis Kit (New England Biolabs) in the presence of Bio−11-UTP (Roche). After bio-ds*Lmβ-tubulin* was quantified by Nanodrop 2000 spectrophotometer, it was diluted with RNase-free water to a concentration of 1 μg/μL. To produce streptavidin-bio-ds*Lmβ-Tubulin* complexes, 2 μL bio-ds*Lmβ-tubulin* (i.e. 2 μg) was incubated with 50 μL of streptavidin beads (Thermo Fisher, Waltham, MA, USA) for 2 h at 4°C with gentle agitation. After the beads were washed with 1× binding and washing buffer (1 × BW buffer: 5 mM Tris-HCl, 0.5 mM EDTA, and 1 M NaCl; pH = 7.5) for three times to remove free bio-ds*Lmβ-tubulin*.

*In vitro* binding assay of LmLoqs protein was performed as follows: For the treatment group, 200 μg LmLoqs-PA/-PB/-PC or LmLoqs-PA-mut supernatant protein was incubated with streptavidin-bio-ds*Lmβ-Tubulin* complex overnight at 4°C, whereas the negative controls were set up with the same amount of the pET32a supernatant protein. To inhibit the degradation of protein or dsRNA, a protease inhibitor cocktail (MedChemExpress, Monmouth Junction, NJ, USA) and RNase inhibitor (TaKaRa) were added to 1× BW buffer. The affinity matrix was then washed five times by magnet with 1× BW buffer to purify the protein that can stably bind to dsRNA. Western blotting was used to confirm that the anti-LmLoqs polyclonal antibody interacted with the target protein in the sample.

### 2.8. Expression analysis of small RNA

To discover the function of *LmLoqs* in endo-siRNA and miRNA RNAi pathways in *L. migratoria*, 1-day-old third-instar nymphs were selected and 3 μg of *LmLoqs* dsRNA was injected as described in Section 2.4. The control group was injected with the same amount of ds*GFP*. The expression of endo-siRNAs [20] and miRNAs was analysed by RT-qPCR using specific primers (Table S3). The small RNA was extracted using Trizol, and miRNA or siRNA was obtained by reverse transcription using the miRcute Plus miRNA First-Strand cDNA Kit (Tiangen, Beijing, China). RT-qPCR was performed using miRcute Plus miRNA qPCR Kit (Tiangen, Beijing, China).

### 2.9. Expression analysis of miRNA in S2 cell

To further study which isoform was involved in miRNA pathway, 1 μg constructed plasmids of pAc5.1-LmLoqs-PA/, -PB/, -PC, or the control group pAc5.1-Lmβ-Tubulin was transferred into S2 cells. The Lipofectamine® 3000 was used as a transfection reagent. After 48 h of transfection, S2 cells were collected and the cell culture medium was discarded. After the S2 cells were washed twice with 1× PBS (137 mM NaCl, 2.7 mM KCl, 4.3 mM Na_2_HPO_4_, 1.4 mM K_2_HPO_4_, PH = 7.4), total RNA enriched with small RNA was extracted as described above. Each experimental group and control group were replicated for 4 times. The miRNAs of S2 cells were quantified using RT-qPCR as described in Section 2.8 [21]. Specific quantitative primers are shown in Table S3.

### 2.10. Statistical analysis

The relative expression of *LmLoqs* was standardized by internal reference gene and analysed using the 2^−ΔΔCt^ method [22]. The data of *LmLoqs* expression in different tissues and times as well as the data of dual-luciferase experiment in S2 cells were analysed by one-way ANOVA followed by Tukey’s honestly significant difference (HSD) test (*p* < 0.05) using SPSS15.0 software (SPSS Inc., Chicago, IL, USA). For the data from the RNAi of RNAi experiment, the percentage data of relative gene expression were transformed by arcsine square root transformation method followed by Tukey’s HSD test. The data from the RNAi silencing efficiency assays were analysed using Student’s *t*-test.

## 3. Results

### 3.1. Molecular characteristics and expression analysis of *LmLoqs*

**Figure 1.**
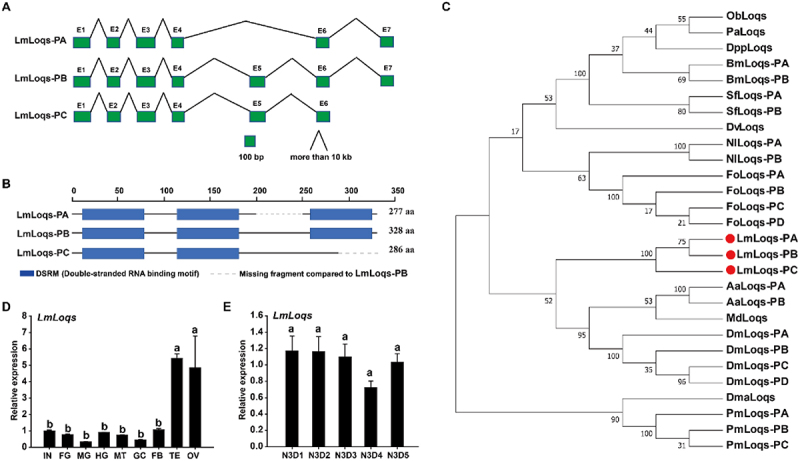
Bioinformatics analysis and expression pattern of LmLoqs in L. migratoria. (A) Schematic diagram of the exon-intron organizations of LmLoqs-PA/-PB/-PC. Green solid boxes represent the exons and black broken lines represent the introns. (B) Domain architecture of deduced amino acid sequences of LmLoqs-PA/-PB/-PC from L. migratoria. Blue rectangles represent DSRM (dsRNA binding motif) domains. The dotted grey line represents the missing fragment compared to LmLoqs-PB. (C) a phylogenetic tree constructed with the neighbour-joining method using MEGA 7.0 software. Bootstrap support was based on 1000 reassembled data sets. Species names and abbreviations and GenBank accession numbers for their respective Loqs proteins are listed in Table S2. (D) Expression analysis of LmLoqs transcript in different tissues dissected from the 3-day-old third-instar (N3D3) nymphs by RT-qPCR, Integument (IN), foregut (FG), midgut (MG), hindgut (HG), Malpighian tubules (MT), gastric caeca (GC), fat bodies (FB), testis (TE) and ovary (OV). (E) Expression analysis of LmLoqs transcript in different days of the whole body of the 1-day- to 5-day-old third-instar (N3D1-N3D5) nymphs by RT-qPCR. All data are reported as mean ± standard error of three independent biological replicates. Different letters on the standard-error bars represent significant differences (*p* < 0.05, Tukey’s HSD test; *n* = 3).

In *L. migratoria*, we revealed three distinct alternative splicing variants of Loqs mRNA as dsRBP isoforms named Loqs-PA, -PB and -PC. The obtained full-length cDNA sequences were further aligned with the *L. migratoria* genome, and their gene structures were deduced (Figure 1A). Results showed that Lmloqs-PB contained seven exons and six introns. LmLoqs-PA lacked the fifth exon and LmLoqs-PC lacked the seventh exon compared with the LmLoqs-PB. Sequence analysis revealed that LmLoqs-PA, LmLoqs-PB, and LmLoqs-PC consisted of 277, 328, and 286 amino acid residues, respectively (Figure 1). Domain analysis showed that LmLoqs-PA and LmLoqs-PB contained three dsRNA binding motif (DSRM) domains, whereas LmLoqs-PC had two DSRM domains (Figure 1B). Phylogenetic analysis showed that the three alternative splicing variants of LmLoqs can be grouped into one cluster with the highest homology (Figure 1C).

We also determined the tissue- and development-specific expression patterns of *LmLoqs* by RT-qPCR. The expression of the *LmLoqs* transcripts was high in the testis and ovary but low in other tissues including the integument, foregut, midgut, hindgut, Malpighian tubules, gastric caecum, and fat body dissected from the N3D3 nymphs (Figure 1D). However, *LmLoqs* showed stable expression in 1-day to 5-day-old third-instar nymphs (Figure 1E).

### 3.2. LmLoqs-PA and LmLoqs-PB are necessary for the exo-siRNA pathway

**Figure 2.**
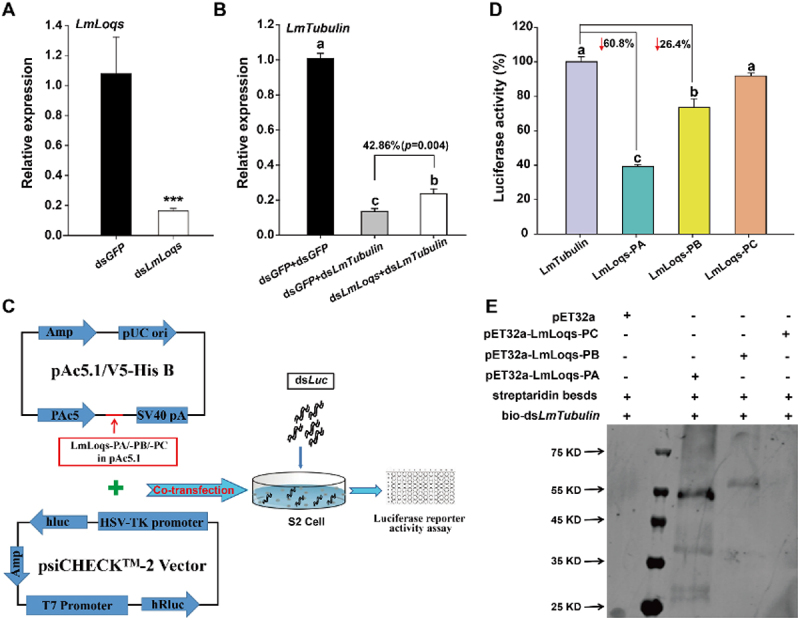
Functional analysis of LmLoqs in exogenous siRNA-mediated RNAi pathway. (A) Analysis of RNAi efficiency against LmLoqs in third-instar L. migratoria (mean±SE, *n* = 4). (B) Effect of RNAi against each LmLoqs on the efficiency of RNAi against the target gene Lmβ-Tubulin in the third-instar L. migratoria (mean ±SE, *n* = 6). (C) Diagram of the double luciferase experimental setup. The pAc5.1 constructs expressing LmLoqs-PA/PB/PC or Lmβ-Tubulin and psicheck − 2 plasmid were used to cotransfect S2 cells. After 4 h, 2 μg dsluc was added to the medium, and fluorescence was measured after 48 h. Two genes encoding Firefly luciferase and Renilla luciferase are presented in psicheck − 2, and dsLuc can interfere with the transcripts of Firefly luciferase. Therefore, we used the ratio of firefly/Renilla to determine whether over-expression of LmLoqs-PA/-PB/-PC proteins in S2 cells can affect RNAi efficiency. (D) Effect of over-expression of LmLoqs-PA/PB/PC on RNAi efficiency in S2 cells (mean ± SE, *n* = 4). (E) Binding analysis of each LmLoqs protein with dsRNA in vitro. The LmLoqs-PA and LmLoqs-PB proteins enriched the formation of streptavidin-bio-dsLmβ-Tubulin complex as detected by western blotting. The silencing efficiency was analysed using Student’s t-test, and *** *p* < 0.001. The RNAi of RNAi data and luciferase activities are analysed using one-way analysis of variance followed by Tukey’s honestly significant difference test (*p* < 0.05). Different letters on the standard-error bars represent significant differences in the expression among the treatments.

The RNAi of RNAi experiments showed that the transcript level of *LmLoqs* was suppressed by 91.4% after injection of ds*LmLoqs* compared with that of the control group (Figure 2A). Meanwhile, compared with the control group, the expression of the target gene *Lmβ-Tubulin* was up-regulated by 42.9% after the suppression of *LmLoqs* (Figure 2B). To verify this result, we included another target gene (*LmLgl*) in our analysis. Our results also showed that the efficiency of RNAi against *LmLgl* was also reduced compared to the control (Fig. S2).

Due to the high sequence similarity of the three alternative splicing variants of *LmLoqs*, we distinguished the functions of the three variants in siRNA pathway with luciferase experiment in *Drosophila* S2 cells (Figure 2C). Our results showed that LmLoqs-PA and LmLoqs-PB enhanced the RNAi efficiency of S2 cells by 60.5% and 26.3%, respectively, whereas LmLoqs-PC did not enhance the RNAi efficiency of S2 cells as compared with the control group (Figure 2D). After we verified the specificity of the LmLoqs antibody (Fig. S3), we used western blotting to confirm that the recombinant plasmid was successfully expressed in S2 cells, which further verified our results from the luciferase assays (Fig. S4).

Furthermore, we successfully expressed the active proteins of LmLoqs-PA, LmLoqs-PB and LmLoqs-PC (Fig. S5) and used these proteins to bind dsRNA *in vitro*. Our results showed that LmLoqs-PA and LmLoqs-PB can bind the dsRNA *in vitro*, but LmLoqs-PC cannot (Figure 2E).

### 3.3. LmLoqs-PB and LmLoqs-PC are involved in the endo-siRNA pathway

**Figure 3.**
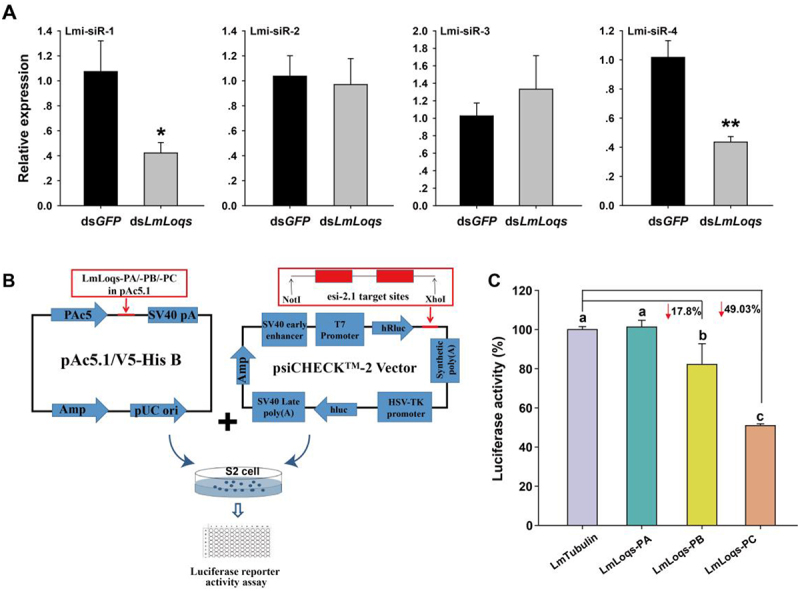
Functional analysis of LmLoqs in endogenous siRNA-mediated RNAi pathway. (A) Expression analysis of endo-siRNA transcripts after silencing of LmLoqs in L. migratoria (mean ±SE, *n* = 4). The endogenous siRNA in L. migratoria were named as Lmi-siR −1/−2/−3/−4 according to Wei et al. [20]. (B) Diagram of Dual-Luciferase reporter assay system. pAc5.1 constructs expressing LmLoqs-PA/-PB/-PC or Lmβ-Tubulin were used to transfect S2 cells. After 4 h, 0.5 μg psicheck2-esi −2.1 plasmid was transfected into the medium, and fluorescence was measured after 40 h. (C) Decreased luciferase activity when LmLoqs-PB and LmLoqs-PC were over-expressed in S2 cells in comparison with the control cells transfected with pAc5.1-Lmβ-Tubulin only (mean±SE, *n* = 4). The RT-qPCR was analysed using Student’s t-test, and *, *p* < 0.05; **, *p* < 0.01. The luciferase activities were analysed using one-way analysis of variance followed by Tukey’s honestly significant difference test (*p* < 0.05); different letters on the standard-error bars represent significant differences in the expression among the treatments.

Our results showed that suppression of *LmLoqs* decreased the transcript level of some endogenous siRNAs (endo-siRNA1 and endo-siRNA4) in *L. migratoria* compared with the control group (Figure 3A). To further identify which of the three LmLoqs isoforms was required for endo-siRNA pathway, we over-expressed LmLoqs-PA, LmLoqs-PB and LmLoqs-PC in S2 cells, and then transferred psi-check2-esi−2.1 (containing esi−2.1 target gene fragments) into S2 cells. PAC5.1-LmLoqs-PA/-PB/-PC plasmids and psi-check2-esi−2.1 were used to co-transfect S2 cells (Figure 3B). Because esi−2.1 presented in cultured S2 cells, luciferase would be repressed by esi−2.1 mediated RNA silencing. Our data showed that LmLoqs-PB and LmLoqs-PC can significantly influence the ratio of luciferase/firefly luciferase for hindering the expression of esi−2.1 (Figure 3C). These results indicated that LmLoqs-PB and LmLoqs-PC could affect the endo-siRNA expression in S2 cells.

### 3.4. LmLoqs-PB plays an important role in miRNA pathway

**Figure 4.**
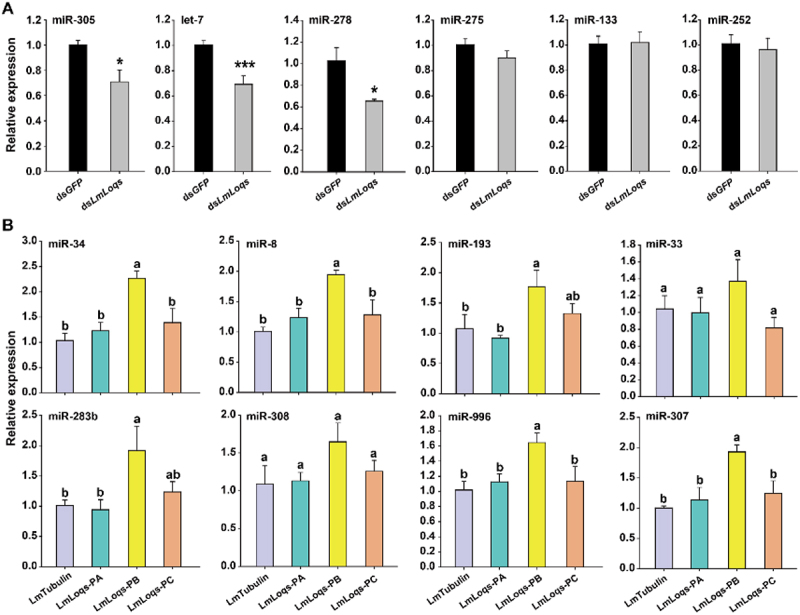
Functional analysis of LmLoqs in the miRNA-mediated RNAi pathway. (A) Effect of the RNAi against LmLoqs on the relative transcript level of each miRNA as determined by RT-qPCR in L. migratoria (mean ±SE, *n* = 4). (B) Effect of the overexpression of each LmLoq on the production of eight miRnas in S2 cells (mean±SE, *n* = 4). The RT-qPCR is analysed using Student’s t-test (*, *p* < 0.05; ***, *p* < 0.001). The data for luciferase activities were analysed using one-way analysis of variance followed by Tukey’s honestly significant difference test (*p* < 0.05). Different letters on the standard-error bars represent significant differences in the expression among the treatments.

Our results showed that knock-down of *LmLoqs* can significantly suppress some miRNAs, such as miR−305, let−7 and miR−278, compared to the control group (Figure 4A), which implied that *LmLoqs* influenced the formation of mature miRNAs in *L. migratoria*. In order to determine the role of different *LmLoqs* variants in the miRNA pathway, we over-expressed LmLoqs-PA, LmLoqs-PB, and LmLoqs-PC in S2 cells, and then tested the expression of miRNAs in S2 cells. Our results showed that only LmLoqs-PB increased the relative expression of miRNAs (miR−34/8/193/283b/996/307) of S2 cells compared with the control (Figure 4B). These results demonstrated that LmLoqs-PB played an important role in the miRNA pathway.

### 3.5. LmLoqs-PA requires the C-terminal 42 residues to bind exogenous dsRNA

**Figure 5.**
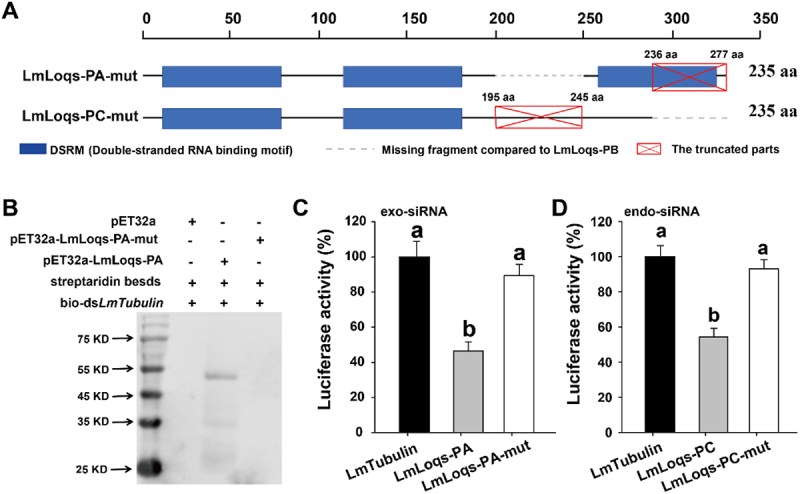
Influence of RNAi efficiency by truncated Lmloqs-PA/PC in S2 cells. (A) Schematics of truncated LmLoqs-PA and LmLoqs-PC. DSRMs are shown as the blue boxes, and the truncated parts are marked by red boxes with crosses. The grey dot line represents the missing fragment compared to LmLoqs-PB. (B) Binding analysis of LmLoqs-PA and truncated LmLoqs-PA (LmLoqs-PA-mut) protein with dsRNA in vitro. The LmLoqs-PA but not the LmLoqs-PA-mut enriched the formation of streptavidin-bio-dsLmβ-Tubulin complex as detected by western blotting. (C) Effect of the overexpression of LmLoqs-PA and LmLoqs-PA-mut proteins on the efficiency of the exogenous siRNA-mediated RNAi in S2 cells (mean±SE, *n* = 4). (D) Effect of the overexpression of LmLoqs-PC and LmLoqs-PC-mut proteins on the efficiency of the endogenous siRNA-mediated RNAi in S2 cells (mean ± SE, *n* = 4). The luciferase activities were analysed using one-way analysis of variance followed by Tukey’s honestly significant difference test (*p* < 0.05). Different letters on the standard-error bars represent significant differences in the expression between the treatments.

To determine which domains of LmLoqs-PA were required to bind exogenous dsRNA in *vitro*, we deleted 42 amino acid residues (236–277 aa) from the C-terminal in comparison with LmLoqs-PC, which disrupts the third DSRM domain of LmLoqs-PA. This new construct was named LmLoqs-PA-mut (Figure 5A and Fig. S1). We also used the dsRNA binding assay and luciferase assay to analyse the role of LmLoqs-PA-mut in exo-siRNA pathway. We successfully expressed the LmLoqs-PA-mut protein (Fig. S6A) and used the protein to bind dsRNA *in vitro*. Our results showed that the LmLoqs-PA-mut protein was not able to bind the dsRNA (Figure 5B). Further, when the sequence of LmLoqs-PA-mut was inserted into the expression vector pAc5.1, LmLoqs-PA-mut did not increase the RNAi efficiency of S2 cells in our luciferase assays (Figure 5C).

### 3.6. LmLoqs-PC requires the intermediate 51 residues to bind endogenous dsRNA

To determine which domains of LmLoqs-PC were required to bind endogenous dsRNA in *vitro*, we deleted 51 amino acid residues (195–245 aa) within the sequence in comparison with LmLoqs-PA, which was named LmLoqs-PC-mut (Figure 5A and Fig. S1). We also used the luciferase assay to analyse the role of LmLoqs-PC-mut in endo-siRNA pathway. Our data showed that LmLoqs-PC-mut cannot influence the ratio of luciferase *Renilla*/firefly luciferase for hindering the expression of esi−2.1 (Figure 5D), indicating that LmLoqs-PC-mut cannot affect the endo-siRNA expression in S2 cells.

## 4. Discussion

Over the past few years, substantial evidence has accumulated that the dsRNA-binding protein Loqs acts as a hub protein in *D. melanogaster* and has four different isoforms that functionally segregate siRNA pathway and miRNA pathway [23,24]. In this study, through repeated search of the *L. migratoria* genome and PCR amplification verification, we found three alternative splicing variants of *Loqs* in *L. migratoria*. In comparison with the four Loqs identified in *D. melanogaster*, Loqs-PD was missing in *L. migratoria*. Although both *A. aegypti* and *D. melanogaster* belong to dipterans, *A. aegypti* also lacks Loqs-PD, suggesting that the occurrence of Loqs-PD may not be conserved even in closely related insect species [16].

The siRNA-mediated RNAi pathway can be further divided into exo- and endo-RNAi pathways according to different sources of dsRNA. To analyse exo-dsRNA-mediated RNAi pathway, we carried out the RNAi of RNAi assays to study the function of *LmLoqs* in *L. migratoria*. Our results indicated that suppression of *LmLoqs* expression can suppress the RNAi efficiency as compared with the control, which implies that *LmLoqs* can participate in exo-RNAi pathway thus affecting RNAi efficiency. These results are consistent with earlier research in *Bombyx mori*, which shows that BmLoqs-depleted cells displayed lower RNAi efficiency than that of the control, indicating that Loqs can affect the RNAi efficiency [25]. However, different isoforms of BmLoqs have not been studied in detail and their functions in the exo-siRNA pathway have not been elucidated in *B. mori*. By using luciferase assays *in vitro*, our current study showed that over-expression of LmLoqs-PA and LmLoqs-PB in S2 cells improved the RNAi efficiency. At the same time, our *in vitro* binding assays showed that LmLoqs-PA and LmLoqs-PB proteins could bind the exogenous dsRNA, which further supports our hypothesis that LmLoqs can participate in the exo-siRNA pathway.

In *Caenorhabditis elegans*, the Loqs-related RDE−4 protein (E-value = 0.03) preferentially binds to long dsRNA (e.g. 650 bp), which leads to the cleavage of dsRNA into siRNA [26]. Previous studies also showed that BmLoqs and BmDicer−2 were co-localized to some specific cytoplasmic foci in *Bombyx mori* cells [25]. Both results support our finding that *LmLoqs* play an important role in exo-siRNA pathway in *L. migratoria*. Gao et al. [7] found another double-stranded binding protein LmR2D2 and showed its ability to bind dsRNA *in vitro*. Indeed, Loqs and R2D2 are known to act sequentially in the siRNA pathway in *D. melanogaster* [14]. Accordingly, our results suggest that LmLoqs-PA/-PB may play a role in the initiation step of the siRNA pathway mediated by exogenous dsRNA in *L. migratoria*.

In *D. melanogaster*, the loss of Loqs greatly reduced the siRNA level generated from the endo-siRNAs mediated RNAi pathway in S2 cells [27]. In present study, we found the expression of endo-siRNAs (Lmi-siR−1 and Lmi-siR−4) decreased when *LmLoqs* gene was silenced by RNAi, suggesting that *LmLoqs* may involve in endo-siRNA pathway. To understand the roles of *LmLoqs* in endo-siRNA pathway, psi-check2-esi−2.1 was constructed to determine which *LmLoqs* influenced in endo-siRNA mediated RNAi pathway. Esi−2.1, also termed hp-CG4068B, is one of the hp-CG4068 endo-siRNAs [24,28]. Consistent with previous results, our data showed LmLoqs-PB and LmLoqs-PC can affect the abundance of esi−2.1 in S2 cell. These findings suggest that LmLoqs-PB and LmLoqs-PC can participate in endo-siRNA pathway. These results support the idea of the involvement of LmLoqs in endo-siRNA (i.e. endo-siRNAs derived from transposons) mediated RNAi pathway, but its detailed mechanism remains to be elucidated. Haac et al. [16] found that over-expression of Loqs-PA increased the effectiveness of endo-siRNA silencing in mosquitoes. In *Drosophila*, DmLoqs-PD associated with Dicer−2 is required to process a subset of RNA substrates, such as hairpin RNAs, into endo-siRNAs in siRNA-mediated RNAi pathway [11,24,29].

As one of the insect RNAi pathways, miRNA-mediated RNAi pathway can also silence the target gene by degrading the target mRNA [29]. Previous studies demonstrated that Loqs normally functions together with Dicer−1 in miRNA biogenesis. For example, in *Drosophila*, DmLoqs-PB is critical for miRNA pathway, whereas in *Drosophila* S2 cells, depletion of Loqs results in pre-miRNA accumulation [5]. In *A. aegypti*, Loqs-PB participates in miRNA biogenesis and this isoform appears to significantly antagonize siRNA production [16]. Consistently, we found that the expression of some miRNAs was suppressed after *LmLoqs* was silenced in *L. migratoria*. To further clarify if LmLoqs was required in miRNA-mediated RNAi pathway, we overexpressed individual LmLoqs-PA, LmLoqs-PB, and LmLoqs-PC in S2 cells, and found that LmLoqs-PB can enhance the production of miRNAs in S2 cells. Taken together, our results have proven that LmLoqs-PB plays an important role in biogenesis of miRNA in *L. migratoria*. Thus, we conclude that Lmloqs-PB can participate in both miRNA- and siRNA-mediated RNAi pathways in *L. migratoria*, which is similar to the situations where LmAgo1, LmAgo2a, LmAgo2b and LmAgo3 each makes a significant contribution to RNAi efficiency in *L. migratoria* [30]. Because LmLoqs-PB is the only LmLoqs containing all the seven exons among the three alternative splicing variants, the structure of LmLoqs-PB may allow its participations in both siRNA- and miRNA-mediated RNAi pathways in insects.

Indeed, the structural integrity of LmLoqs-PA and LmLoqs-PC played an important role in the exogenous and endogenous siRNA-mediated RNAi pathways, respectively. When the third dsRBD domain was disrupted by truncating the 42 amino acid residues at the C-terminal of LmLoqs-PA, the structurally modified LmLoqs-PA lost its ability to bind exogenous dsRNA. When the 51 amino acid residues of the non-dsRBD domain in the middle of LmLoqs-PC was deleted, LmLoqs-PC was also not able to bind endogenous dsRNA. We hypothesize that the modified Lmloqs-PC might have lost its structural integrity, which prevents its binding to endogenous dsRNA. The structurally modified LmLoqs-PC might have also failed to bind Dicer−2, which prevents the cleavage of the endogenous dsRNA by Dicer−2. In *Drosophila*, the C-terminal 22 amino acid residues of Loqs-PD are found to be essential for the binding of Loqs-PD to Dicer−2, and this binding is independent of the presence of dsRNA [12]. In mosquitoes, however, the junction between the second and third dsRBD domains of Loqs-PA does not play a role in Dicer binding [16]. Further research is needed to identify the specific-binding sites of LmLoqs-PC for endogenous dsRNA and for Dicer−2 in *L. migratoria*.

**Figure 6.**
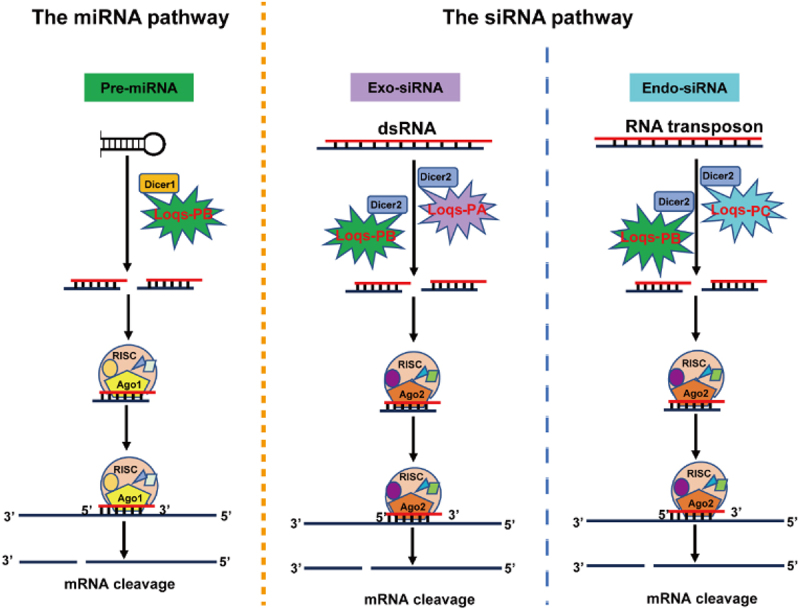
The model illustrating the roles of Loqs proteins in different RNAi pathways of L. migratoria. In the miRNA-mediated RNAi pathway, Loqs-PB assists the binding between pre-miRNA and Dicer−1, which results in the cleavage of pre-miRNA to yield matured miRNA. In the siRNA-mediated RNAi pathways, there are two different mechanisms depending on the sources of dsRNA. In exogenous siRNA-mediated RNAi pathway, binding of Loqs-PA or LmLoqs-PB to exogenous dsRNA facilitates the cleavage of dsRNA by Dicer−2, whereas in endogenous siRNA-mediated RNAi pathway, binding of Loqs-PB or Loqs-PC to endogenous dsRNA facilitates the cleavage of dsRNA by Dicer−2.

Based on our results, we propose a model in which Loqs mediates small RNA pathway in *L. migratoria* (Figure 6). In the miRNA-mediated RNAi pathway, Loqs-PB assists the binding between pre-miRNA and Dicer−1, which results in the cleavage of pre-miRNA to yield matured miRNA. In the siRNA-mediated RNAi pathways, there are two different mechanisms depending on the sources of dsRNA. In exogenous siRNA-mediated RNAi pathway, binding of Loqs-PA or Loqs-PB to exogenous dsRNA (e.g. dsRNA delivered by injection) facilitates the cleavage of dsRNA by Dicer−2 to generate exogenous siRNA, whereas in endogenous siRNA-mediated RNAi pathway, binding of Loqs-PB or Loqs-PC to endogenous dsRNA (e.g. RNA transposon) facilitates the cleavage of dsRNA by Dicer−2 to generate endogenous siRNA. This model shows that different dsRNA binding proteins participate in different RNAi pathways for the biogenesis of siRNA. However, further research is needed to develop novel strategies for improving RNAi efficiency by manipulating the biogenesis of siRNA in insects.

## Supplementary Material

Supplemental Material

## Data Availability

Data will be made available on request.
