# Supplementary material for: Three alternative splicing variants of Loquacious play different roles in miRNA- and siRNA-mediated RNAi pathways in Locusta migratoria
Source: RNA Biol. 2023 Jun 13;20(1):323–33. doi: 10.1080/15476286.2023.2223484 (PMC10266119; doi:10.1080/15476286.2023.2223484)
Supplement: Supplemental Material [file KRNB_A_2223484_SM7482.pdf]

Supplemental Data

Three alternative splicing variants of *Loquacious* play different roles in miRNA- and siRNA-mediated RNAi pathways in *Locusta migratoria*

Yanli Wang, Huiyong Li, Xiaojian Liu, Lu Gao, Yunhe Fan, Kun Yan Zhu, and Jianzhen Zhang

Table S1. Primers for full-length cDNA validation, PCR amplification and dsRNA synthesis

**Application of primers**	**Primer names**	**Primer sequence（5’-3’）**	**Products (bp)**
Full length verification	*LmLoqs-PA*	F:ATGGCAGCAGCTGCAGCAGT<br>R:TCACTTTTTTGATAAAATCTTGAGAT	834
	LmLoqs-PB	F:ATGGCAGCAGCTGCAGCAGT<br>R:TCACTTTTTTGATAAAATCTTGAGAT	987
	LmLoqs-PC	F:ATGGCAGCAGCTGCAGCAGT<br>R:CTAACTCTTACCTGTGACAGAC	861
RT-qPCR analysis	*LmLoqs*	F:AAGGATTACCTCATGAACGG<br>R:TCATCGTCTGGTACTGGAG	172
	Lmβ-Tubulin	F:GAAATGGAGTTCACGGAAGC<br>R:CTTGCTCCTCATCAAACTCG	109
	LmLgl	F:ATCATCAGCAGCAGTGAAACAA<br>R:ATTAGAGTCAGGCGGCAAGATG	221
	LmEF1α	F:AGCCCAGGAGATGGGTAAAG<br>R:CTCTGTGGCCTGGAGCATC	155
dsRNA synthesis	*Lmβ-Tubulin*	F:taatacgactcactatagggAGGCCACTACACAGAGGGTG<br>R:taatacgactcactatagggTGACGCCAGACATGGTAAGA	401
	LmLoqs	F:taatacgactcactatagggCAGTGGGGAAGACTCCTG<br>R:taatacgactcactatagggCTTCATGGACCATTTCATAAT	390
	LmLgl	F:taatacgactcactatagggCTGACTTACGGAGGCAGCTC<br>R:taatacgactcactatagggGTAACACGGCCATCCTCTGT	539
	GFP	F:taatacgactcactatagggCACAAGTTCAGCGTGTCCG<br>R:taatacgactcactatagggGTTCACCTTGATGCCGTTC	402

Table S2. Species names and GenBank accession numbers of the deduced protein sequences used for constructing the phylogenetic tree presented in Figure 1C

Species	Gene name	GenBank accession number
Penaeus monodon	*PmLoqs-PA*	XP_037800866.1
Penaeus monodon	*PmLoqs-PB*	XP_037800867.1
Penaeus monodon	*PmLoqs-PC*	XP_037800868.1
Danaus plexippus plexippus	*DppLoqs*	OWR41099.1
Daphnia magna	*DmaLoqs*	KZS09510.1
Operophtera brumata	*ObLoqs*	KOB76149.1
Bombyx mori	*BmLoqs-PA*	XP_012550851.2
Bombyx mori	*BmLoqs-PB*	XP_021207535.2
Pararge aegeria	*PaLoqs*	JAA88426.1
Mayetiola destructor	*MdLoqs*	AFX89033.1
Nilaparvata lugens	*NlLoqs-PA*	XP_022189835.2
Nilaparvata lugens	*NlLoqs-PB*	XP_022189837.2
Aedes aegypti	*AaLoqs-PA*	AJF11546.1
Aedes aegypti	*AaLoqs-PB*	AJF11545.1
Drosophila melanogaster	*DmLoqs-PA*	NP_723813.1
Drosophila melanogaster	*DmLoqs-PB*	NP_609646.1
Drosophila melanogaster	*DmLoqs-PC*	NP_001033903.1
Drosophila melanogaster	*DmLoqs-PD*	NP_001188796.1
Diabrotica virgifera	*DvLoqs*	AVK59433.1
Spodoptera frugiperda	*SfLoqs-PA*	AVK59447.1
Spodoptera frugiperda	*SfLoqs-PB*	AVK59446.1
Frankliniella occidentalis	*FoLoqs-PA*	KAE8750710.1
Frankliniella occidentalis	*FoLoqs-PB*	KAE8750711.1
Frankliniella occidentalis	*FoLoqs-PC*	KAE8750709.1
Frankliniella occidentalis	*FoLoqs-PD*	KAE8750712.1

Table S3. Primer sequences used to analyze the expression of small RNA

Primer names	Primer sequence
miRNA-305	F:ACACTCCAGCTGGGATTGTACTTCATCA
	R:CTCAACTGGTGTCGTGGAGTCGGCAA
Let-7	F:ACACTCCAGCTGGGTGAGGTAGTAGGT
	R:CTCAACTGGTGTCGTGGAGTCGGCAA
miRNA-278	F:ACACTCCAGCTGGGTCGGTGGGACTTTCGT
	R:CTCAACTGGTGTCGTGGAGTCGGCAA
miRNA-275	F:ACACTCCAGCTGGGTCAGGTACCTGAAGTA
	R:CTCAACTGGTGTCGTGGAGTCGGCAA
miRNA-133	F:ACACTCCAGCTGGGTTTGGTCCCCTTCAACC
	R:CTCAACTGGTGTCGTGGAGTCGGCAA
miRNA-252	F:ACACTCCAGCTGGGCTAAGTACTAGTGCCG
	R:CTCAACTGGTGTCGTGGAGTCGGCAA
endo-siRNA 1	F:ACACTCCAGCTGGGTTACAACTGTCAGAGCGC
	R:CTCAACTGGTGTCGTGGAGTCGGCAA
endo-siRNA 2	F:ACACTCCAGCTGGGTTAGTTTCAGCACGGACG
	R:CTCAACTGGTGTCGTGGAGTCGGCAA
endo-siRNA 3	F:ACACTCCAGCTGGGTCAGGCACATACAAAACA
	R:CTCAACTGGTGTCGTGGAGTCGGCAA
endo-siRNA 4	F:ACACTCCAGCTGGGTCAGGCACATACAAAAGA
	R:CTCAACTGGTGTCGTGGAGTCGGCAA
XhoI-esi-2.1	F:TCGAGCAACAGTTTATTGGAGCGAACTTGTTGGAGTCAAAATGAACTGAGGGTGG AGCGAACTTGTTGGAGTCAAGC
NotI-esi-2.1	R:GGCCGCTTGACTCCAACAAGTTCGCTCCACCCTCAGTTCATTTTGACTCCAACAAG TTCGCTCCAATAAACTGTTGC

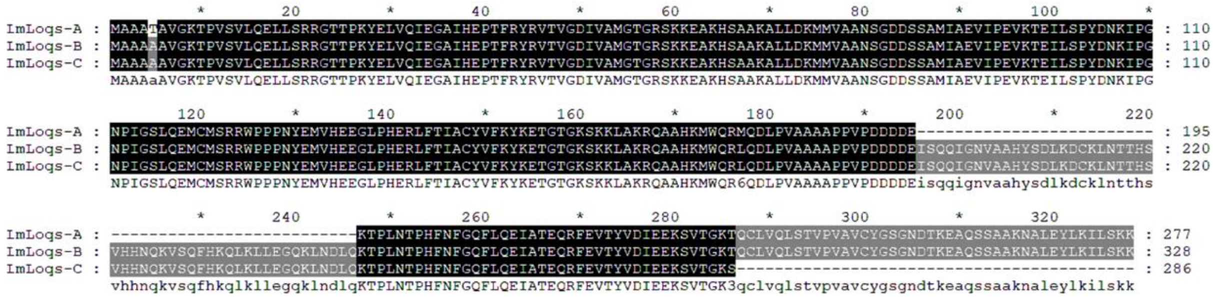

Figure S1. Alignments of amino acid sequences of LmLoqs-PA, LmLoqs-PB and LmLoqs-PC. The sequences boxed with the red dashed lines indicate the truncated parts.

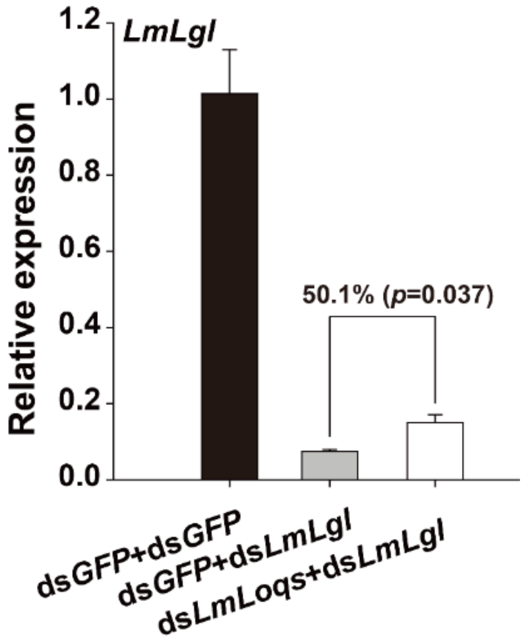

Figure S2. The RNAi of RNAi experiment. Relative transcript levels of the target gene *LmLgl* after silencing of *LmLoqs* in the third-instar *L. migratoria* (mean±SE, n=6). The RNAi of RNAi data were analyzed using one-way analysis of variance followed by Tukey's honestly significant difference test ($p<0.05$); different letters represent significant differences in the expression between the treatments.

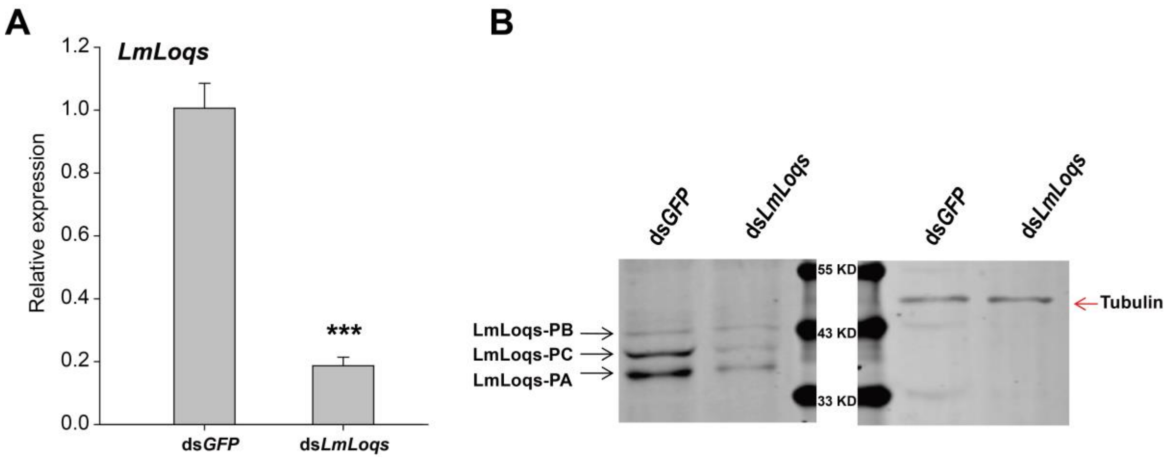

Figure S3. Detection of the three LmLoqs proteins using LmLoqs polyclonal antibody after RNAi against each LmLoqs variant in the 1-day-old third-instar nymphs (N3D1). (A)The efficiency of RNAi against LmLoqs as determined by RT-qPCR. The data are reported as the mean±SE. (B) Detection of LmLoqs proteins after RNAi against LmLoqs gene using western blotting analysis. LmLoqs polyclonal antibody was used in the analysis, whereas β-Tubulin monoclonal antibody was used as the loading control.

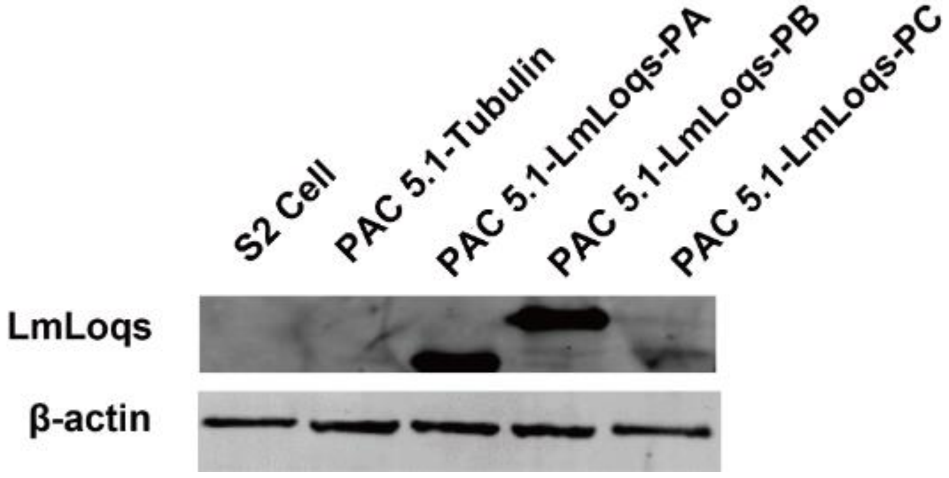

Figure S4. The expression of the recombinant LmLoqs proteins in S2 cells as confirmed by western blotting using the LmLoqs polyclonal antibody.

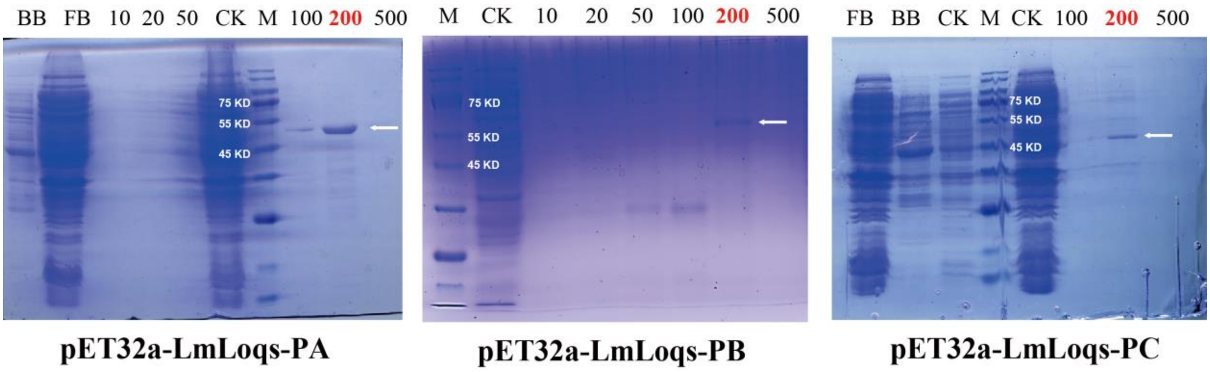

Figure S5. SDS-polyacrylamide gel electrophoresis (SDS-PAGE) gel followed by Coomassie blue staining analysis to show the expression of the LmLoqs-PA, Lmloqs-PB, and LmLoqs-PC proteins. The numbers 10, 20, 50,100, 200,500 represent the proteins eluted by 10, 20, 50,100, 200 and 500 mmol L^{-1} imidazole. M: molecular size markers; FB: flow-through buffer; BB: binding buffer.

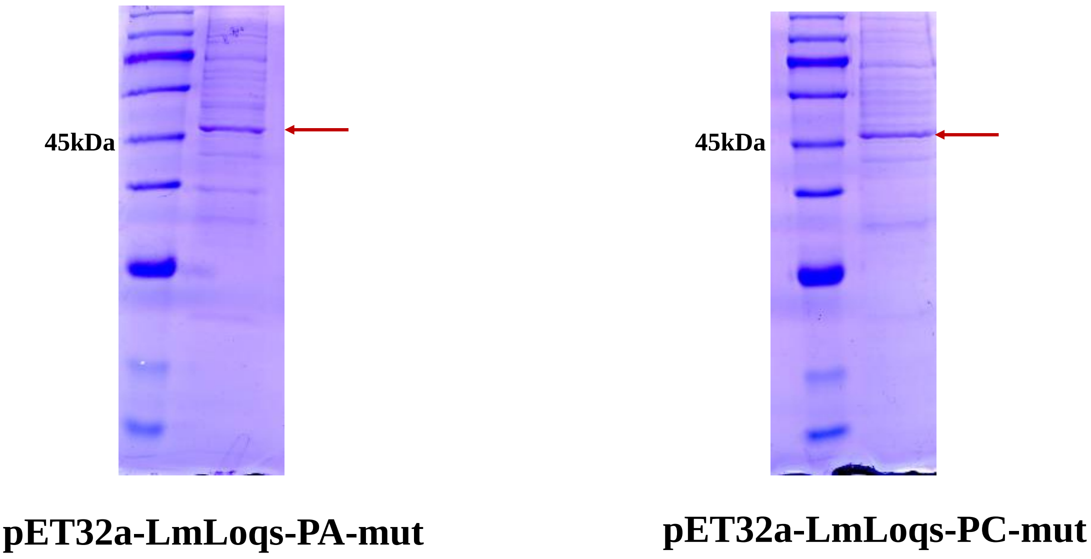

Figure S6. SDS-polyacrylamide gel electrophoresis (SDS-PAGE) gel followed by Coomassie blue staining analysis to show the expression of the LmLoqs-PA-mut and LmLoqs-PC-mut proteins.
